# Development and validation of a predictive model for adverse left ventricular remodeling in NSTEMI patients after primary percutaneous coronary intervention

**DOI:** 10.1186/s12872-022-02831-2

**Published:** 2022-08-27

**Authors:** Lili Wang, Tao Liu, Chaofan Wang, Haochen Xuan, Xianzhi Xu, Jie Yin, Xiaoqun Li, Junhong Chen, Dongye Li, Tongda Xu

**Affiliations:** 1 Department of Cardiology, The Affiliated Hospital of Xuzhou Medical University, Xuzhou, 221000 Jiangsu China; 2 School of Stomatology, Xuzhou Medical University, Xuzhou, 221000 Jiangsu China; 3 Department of General Practice, The Affiliated Hospital of Xuzhou Medical University, Xuzhou, 221000 Jiangsu China

**Keywords:** Non-ST-elevation myocardial infarction, Adverse left ventricular remodeling, Percutaneous coronary intervention, Nomogram model

## Abstract

**Introduction:**

To develop and validate clinical evaluators that predict adverse left ventricular remodeling (ALVR) in non-ST-elevation myocardial infarction (NSTEMI) patients after primary percutaneous coronary intervention (PCI).

**Methods:**

The retrospective study analyzed the clinical data of 507 NSTEMI patients who were treated with primary PCI from the Affiliated Hospital of Xuzhou Medical University and the Second Affiliated Hospital of Xuzhou Medical University, between January 1, 2019 and September 31, 2021. The training cohort consisted of patients admitted before June 2020 (*n* = 287), and the remaining patients (*n* = 220) were assigned to an external validation cohort. The endpoint event was the occurrence of ALVR, which was described as an increase ≥ 20% in left ventricular end-diastolic volume (LVEDV) at 3–4 months follow-up CMR compared with baseline measurements. The occurrence probability of ALVR stemmed from the final model, which embodied independent predictors recommended by logistic regression analysis. The area under the receiver operating characteristic curve (AUC), Calibration plot, Hosmer–Lemeshow method, and decision curve analysis (DCA) were applied to quantify the performance.

**Results:**

Independent predictors for ALVR included age (odds ratio (OR): 1.040; 95% confidence interval (CI): 1.009–1.073), the level of neutrophil to lymphocyte ratio (OR: 4.492; 95% CI: 1.906–10.582), the cardiac microvascular obstruction (OR: 3.416; 95% CI: 1.170–9.970), peak global longitudinal strain (OR: 1.131; 95% CI: 1.026–1.246), infarct size (OR: 1.082; 95% CI: 1.042–1.125) and left ventricular ejection fraction (OR: 0.925; 95% CI: 0.872–0.980), which were screened by regression analysis then merged into the nomogram model. Both internal validation (AUC: 0.805) and external validation (AUC: 0.867) revealed that the prediction model was capable of good discrimination. Calibration plot and Hosmer–Lemeshow method showed high consistency between the probabilities predicted by the nomogram (*P* = 0.514) and the validation set (*P* = 0.762) and the probabilities of actual occurrence. DCA corroborated the clinical utility of the nomogram.

**Conclusions:**

In this study, the proposed nomogram model enabled individualized prediction of ALVR in NSTEMI patients after reperfusion and conduced to guide clinical therapeutic schedules.

## Introduction

More than 7 million patients with new-onset myocardial infarction on a global scale each year, which holds a serious negative impact on human health [1]. Percutaneous coronary intervention (PCI) therapy could open infarct-related vessels in time and reduce short-term mortality significantly [2]. However, numerous patients with myocardial infarction are gradually subjected to adverse left ventricular remodeling (ALVR) after successful reperfusion, afterwards leading to poor outcome events, such as heart failure and even death [3]. Recent studies have demonstrated that patients with non-ST-elevation myocardial infarction (NSTEMI) have a significantly higher risk of adverse events such as late death during follow-up compared with STEMI patients [4, 5]. Moreover, NSTEMI accounts for more than 70% of all myocardial infarction nowadays, and this proportion has been on the rise in recent years [6]. Therefore, early prediction of ALVR is considerable for the interference of therapeutic schedule and the improvement of prognosis in patients with NSTEMI after PCI.

ALVR is a complex process that brings about a range of irreversible changes in the structure and function of the cardiovascular system in response to some physiological and pathological stimuli [7]. At present, the clinical diagnosis of ALVR is mainly based on morphological testing, including the changes in cavity diameter, geometry and scar area after myocardial infarction [8]. In imaging, ALVR is usually characterized as an increase of ≥ 20% in the end-diastolic volume compared with baseline measurement [9], and the conventional detection methods are composed of echocardiography, radionuclidic imaging, and cardiac magnetic resonance (CMR). The imaging quality and measurement accuracy of echocardiography depend on some factors such as acoustic window conditions and the experience of the operator, leading to more subjective results [7]. Radionuclide imaging not only requires the use of radiopharmaceuticals, but also is limited by spatial resolution [10]. CMR has been recognized as the “gold standard” for the non-invasive estimation of cardiac morphology and function owing to its distinguished resolution, accuracy, reproducibility, and non-radioactive feature [11]. Meanwhile, it could provide a “one-stop” examination of the anatomical structure, motor function, myocardial perfusion, and tissue characteristics of the heart, so the correlative indices of CMR were applied to evaluate ALVR in the study. A quite number of projects have been conducted to explore predictors of ALVR, and the results contained circulating biomarkers, angiography, imaging parameters, etc. [12–17]. Note that previous studies on ALVR were mostly confined to ST-elevation myocardial infarction, while NSTEMI were few involved. Furthermore, most of these researches have been concentrated on the analysis of single independent risk factors, and a comprehensive predictive scoring system integrating the predictors has not been established to date.

Nomogram, a graphical form of the clinical prediction model, can calculate the probability of an event quantitatively [18]. As a result of its intuitiveness and accuracy, the nomogram has been broadly applied in medical diagnosis, treatment strategies, and prognosis management [19]. The study aimed at building a risk prediction nomogram based on clinical data of Chinese patients, which can early screen patients at high risk for ALVR events, for timely intervention and treatment to improve the long-term prognosis of patients.

## Material and methods

### Study population and design

This study was conducted by the Declaration of Helsinki and was approved by the Medical Research Ethics Committee of the Affiliated Hospital of Xuzhou Medical University. All clinical information involved for retrospective analysis were available from the Affiliated Hospital of Xuzhou Medical University and the Second Affiliated Hospital of Xuzhou Medical University. 742 consecutive patients with first-onset NSTEMI hospitalized between January 1, 2019 and September 31, 2021 were initially included. Patients who conformed to all the following conditions were collected: (1) Patients were diagnosed with NSTEMI by relevant guidelines and received primary PCI successfully (TIMI grade ≥ 2 after PCI) within 24 h; (2) Patients received cardiac magnetic resonance (CMR) within 7 days after revascularization. Exclusive criteria: (1) Medical history of myocardial infarction or coronary artery bypass grafting; (2) Patients with one of the following complications: severe liver or kidney disfunction, malignant tumour, acute infection, severe haematological disorder, autoimmune disease, congenital heart disease, malignant arrhythmia, rheumatic heart disease, Killip class III or IV; (3) Patients lost to follow-up.

**Fig. 1:**
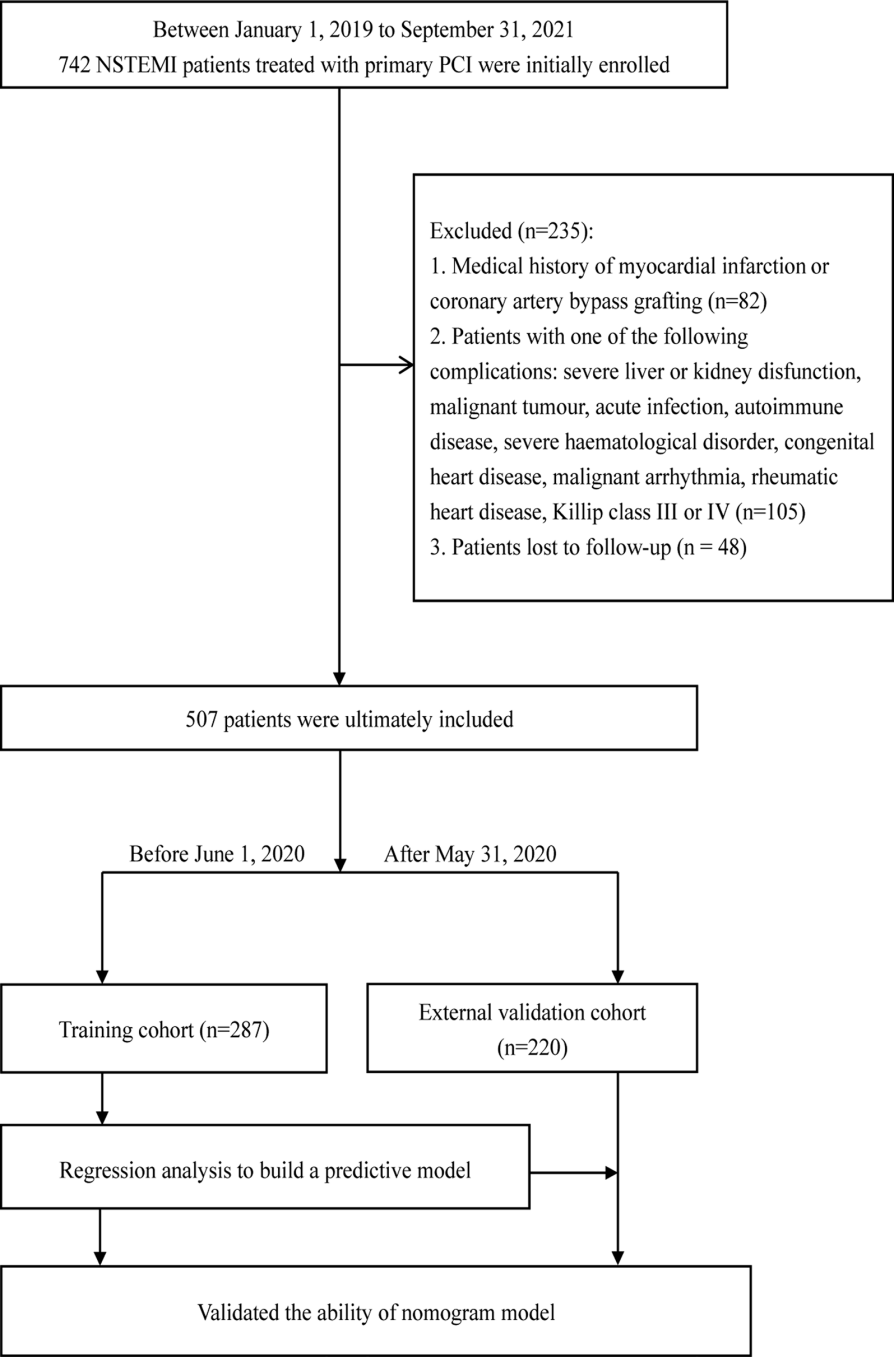
Flow chart. Abbreviations: NSTEMI, non-ST-elevation myocardial infarction; ALVR, adverse left ventricular remodeling

Ultimately, 507 patients were included in this study. 287 patients admitted before June 1, 2020 were assigned to the training cohort, and 220 patients admitted after May 31, 2020 were splitted into the validating cohort. The nomogram was established and validated through the training and validation groups, respectively. Patient enrollment and study design were shown in Fig. 1.

### Treatment strategies during hospitalization

All patients were administered the loading dose of 300 mg aspirin, 600 mg clopidogrel or 180 mg ticagrelor before the procedure, exclusive of those who had received dual antiplatelet therapy (DAPT) one week before the operation. Therapeutic schedule: all patients underwent coronary angiography and then underwent PCI or drug conservative treatment according to the characteristics of coronary lesions and the attitudes of patients’s families. The selection of interventional equipment and adjuvant drugs (nitroprusside, atropine, tirofiban, etc.) were determined by operators on the basis of the specific situation of each patient. After the operation, all patients were treated with DAPT in accordance with the related guidelines, β-blockers, angiotensin-converting enzyme inhibitor/angiotensin receptor blocker, statins, nitrates, aldosterone antagonists and other drugs, as appropriate.

### CMR imaging protocol

CMR imaging was performed with a 3.0-T MRI Scanner (Ingenia 3.0 T, Philips Healthcare, The Netherlands) within 7 days and 3–4 months after primary PCI, equipped with a 5-unit cardiac coil, electrocardiographically-gated technique, and a dual-channel high pressure injector. Patients remained in supine position, and images were captured while the patient was holding his breath at the end of a calm exhalation. Cine Imaging: free steady-state fast gradient echo (BTFE) sequence, continuous scanning from apex of the LV to base and three long-axis planes (2, 3, and 4 chambers) to assess cardiac function and volume. Black Blood Imaging: T2-weighted short-tau inversion recovery (T2-STIR) sequences, scanned covers the entire left ventricular short-axis to evaluate compositional changes in myocardial tissue. Perfusion imaging: Dynamic SENSE-TFE sequences, scanning left ventricular short-axis position while Gd-DTPA (0.15 mmol/kg, 3 mL/s) was injected intravenously. Late gadolinium enhancement (LGE) images were captured 15–20 min after contrast injection through the phase-sensitive inversion recovery (PSIR) sequence. The scanning parameters: echo time 1.4 ms, repetition time 2.30 ms, section thickness 8 mm, the field of view 350 * 350 mm.

### CMR image post-processing

CMR cine images post-processing were performed with CVI software (version 5.13.9, Circle Cardiovascular Imaging Inc, Canada) by two senior radiologists independently. The functional parameters of the left ventricle were obtained by analyzing the Cine sequence (short-axis portion) using the LAX module of the software. Cine images of the short and long axes were analyzed by the Strain module to access strain parameters. Semi-automatic delineation of the LV endocardial and epicardial borders was performed in each section at end-diastole, then manually corrected according to the morphological features if necessary. Peak global strain values were obtained by analysis, including peak global circumferential strain (GCS), peak global radial strain (GRS), and peak global longitudinal strain (GLS). The collected CMR delayed enhancement sequences were analyzed using the Tissue Signal Intensity module. The area of delayed enhancement was showed as the high signal area consistent with the distribution of culprit vessels on the LGE image, that was, an area where the signal intensity is greater than 5 standard deviations (SD) of normal myocardial signal [20]. The total infarct size (IS) was described as the percentage of delayed enhancement area volume to total left ventricular volume [21]. On LGE imaging, the persistent and position invariant low signal region within the high signal region throughout the complete cardiac cycle was defined as cardiac microvascular obstruction (CMVO) [22]. Intramyocardial hemorrhage (IMH) presents as the hypointense region within the hyperintense region on T2 sequences, which was attained by T2 module analysis.

### Follow up and clinical endpoint

By reviewing the electronic medical records, those patients who completed the evaluation of electrolytes, liver and kidney function, electrocardiogram, and CMR within 3–4 months after PCI were considered to be successfully followed up. The clinical endpoint was the occurrence of ALVR, which was described as an increase ≥ 20% in left ventricular end-diastolic volume (LVEDV) at 3–4 months follow-up CMR compared with baseline measurements [23, 24].

### Data collection

Assembled clinical data through electronic medical records, covering demographic information, physical examination data, laboratory parameters, treatment strategies (embracing medication, coronary angiography, and PCI) and correlative indices of CMR.

Demographic information were comprised of sex, age, smoking or drinking history, pre-infarction angina (pre-AP), and Killip class. Physical examination results consisted of body mass index (BMI), systolic blood pressure (SBP), diastolic blood pressure (DBP), and heart rate (HR). The past medical history contained hypertension, diabetes, coronary artery disease (CAD), and a history of stent implantation. Laboratory parameters included hemoglobin (HGB), white blood cell (WBC) count, platelet (PLT) count, Neutrophil to Lymphocyte Ratio (NLR), total cholesterol (CHOL), low-density lipoprotein cholesterol (LDL-C), lipoprotein a, triglycerides (TG), high-density lipoprotein cholesterol (HDL-C), lactate dehydrogenase (LDH), creatine kinase isoenzyme (CKMB), high-sensitivity troponin T (hsTnT), cardiac troponin I (cTnI), N-terminal pro-brain natriuretic peptide (NT-proBNP), serum creatinine (CREA), serum uric acid (UA), hemoglobin A1c (HbA1c), and high-sensitivity C-reactive protein (hsCRP). All baseline laboratory indicators were first results of laboratory test within 24 h of admission. Treatment strategies contained symptom onset-to-balloon time (SBT), Multivascular disease, left anterior descending branch (LAD), left circumflex branch (LCX), right coronary artery (RCA), non-culprit chronic total occlusion (CTO), door-to-balloon time (D2B), the number of stents, no-reflow, and the use drug of atropine, dopamine, tirofiban, and nitroprusside. CMR parameters included LVEDV, left ventricular end-systolic volume (LVESV), IS, cardiac output (CO), left ventricular ejection fraction (LVEF), CMVO, IMH and strain values GCS, GRS, and GLS.

### Statistical analysis

Restricted cubic spline was performed to analyze linear relationships between all candidate continuous variables and ALVR. Categorical data are displayed as the frequency with percentage and checked by chi-square test for comparison. The Continuous indicators that conformed to normal distribution were expressed as mean ± standard deviation (x ± s), using the t-test. Continuous data that did not conform to a normal distribution were expressed as medians (quartile) M (Q1, Q3) and compared by the Mann–Whitney U test. Univariate logistic regression analyses were undertaken to screen the potential risk factors of ALVR in NSTEMI patients who had post-PCI, and the variables with significant differences were further selected into multivariate logistic backward stepwise regression analysis. The nomogram was developed based on the variables whose *P*-values < 0.05 in multivariate logistic regression analysis, and the performance (discrimination, calibration, and the clinical utility) was assessed. We used bootstrap method for internal validation, with resampling times B = 1000. The concordance index (C-index) is a common measure of discrimination, and the area under the curve (AUC) determined by the receiver operating characteristic curve (ROC) was utilized to represent C-index in this study. The calibration plot and Hosmer–Lemeshow method were performed to observe the consistency between the predicted and the actual probabilities. In addition, Decision-curve analysis was applied to appraise the clinical utility. In all analysis, with *P* < 0.05 (two-sided test) as the difference was statistically significant. All data were analyzed by R Studio software (version 4.1.3) and Stata software (version 16.0).

## Results

### Baseline characteristics of training group and validation group

507 NSTEMI patients were involved in our study through screening, then they were assigned to the model for the training group (*n* = 287) and the external validation group (*n* = 220). 143 patients were diagnosed with ALVR by re-examining CMR, including 77 (26.80%) in the training team and 66 (30.00%) in the validation team. The differences in baseline characteristics between the two groups were not statistically significant, which showed good comparability (Table 1).

**Table 1:** Clinical characteristics data in the training and test sets

Characteristics	Training set (*n* = 287)	Validation set (*n* = 220)	*P* value
Age, years	57.0 (50.0–66.0)	57.5 (51.0–65.0)	0.652
Sex			0.162
Male, *n* (%)	239 (83.28)	193 (87.73)	
Female, *n* (%)	48 (16.72)	27 (12.27)	
BMI, kg/m^2^	25.25 (23.14–27.70)	24.98 (22.86–27.68)	0.687
SBP, mmHg	126.0 (115.0–139.0)	127.00 (114.0–139.0)	0.881
DBP, mmHg	80.0 (72.0–90.0)	80.00 (71.0–89.0)	0.484
HR, times/min	80.0 (72.0–89.0)	78.00 (71.0–88.0)	0.385
Smoker, *n* (%)			0.798
No	135 (47.04)	106 (48.18)	
Yes	152 (52.96)	114 (51.82)	
Drinker, *n* (%)			0.592
No	153 (53.31)	112 (50.91)	
Yes	134 (46.69)	108 (49.09)	
Hypertension, *n* (%)			0.659
No	193 (67.25)	152 (69.09)	
Yes	94 (32.75)	68 (30.91)	
Diabetes, *n* (%)			0.276
No	211 (73.52)	171 (77.73)	
Yes	76 (26.48)	49 (22.27)	
History of CAD, *n* (%)			0.728
No	214 (74.56)	167 (75.91)	
Yes	73 (25.44)	53 (24.09)	
*Laboratory parameters*			
WBC, × 10^9^/L	10.00 (8.10–11.90)	9.80 (8.00–11.28)	0.341
NLR, %			0.346
< 4.03	107 (37.28)	71 (32.27)	
4.03–7.68	99 (34.49)	89 (40.45)	
> 7.68	81 (28.22)	60 (27.27)	
HGB, g/L	148.0 (136.0–158.0)	147.00 (136.0–156.0)	0.444
PLT, × 109/L	213.39 ± 52.67	217.52 ± 55.05	0.436
hs-CRP, mg/dL	4.20 (1.40–11.10)	3.70 (1.40–11.10)	0.907
cTnI, ng/L	14.34 (4.70–32.19)	13.27 (4.54–28.26)	0.594
hsTnT, ng/L	2600.0 (1028.0–1566.0)	2506.0 (989.5–5143.8)	0.718
CKMB, ng/L			0.602
≦135.00	200 (69.69)	158 (71.82)	
> 135.00	87 (30.31)	62 (28.18)	
NT-proBNP, pg/mL	826.00 (187.00–2100.14)	837.31 (291.49–2120.85)	0.356
CREA, mmol/L	62.0 (54.0–73.0)	62.0 (54.0–73.0)	0.712
UA, umol/L	304.00 (246.00–365.00)	296.00 (242.25–357.50)	0.410
CHOL, mmol/L	4.33 (3.64–5.05)	4.47 (3.80–5.05)	0.300
TG, mmol/L	1.35 (0.91–2.08)	1.42 (1.03–2.06)	0.137
HDL-C, mmol/L	0.99 (0.83–1.21)	1.04 (0.89–1.19)	0.109
LDL-C, mmol/L	2.77 (2.11–3.36)	2.88 (2.23–3.38)	0.123
Lp(a), mg/L	206.00 (129.00–311.00)	207.50 (144.25–325.50)	0.212
LDH, U/L	565.0 (280.0–864.0)	483.0 (257.0–835.0)	0.384
HbA1c, %	6.10 (5.70–6.70)	6.15 (5.70–6.90)	0.183
*Angiographic features*			
SBT, min	240.0 (140.0–390.0)	243.0 (150.0–372.0)	0.764
D2B, min	64.0 (52.0–84.0)	64.5 (52.0–84.0)	0.875
Pre-AP, *n* (%)			0.573
No	210 (73.17)	156 (70.91)	
Yes	77 (26.83)	64 (29.09)	
Killip class, *n* (%)			0.597
I	256 (89.2)	201 (91.36)	
II	22 (7.67)	15 (6.82)	
III	9 (3.14)	4 (1.82)	
History of stent implant, *n* (%)			0.121
No	268 (93.38)	197 (89.55)	
Yes	19 (6.62)	23 (19.45)	
Multivascular disease, *n* (%)			0.785
No	113 (39.37)	84 (38.18)	
Yes	174 (60.63)	136 (61.82)	
LAD, *n* (%)			0.517
No	143 (49.83)	116 (52.73)	
Yes	144 (50.17)	104 (47.27)	
LCX, *n* (%)			0.463
No	248 (86.41)	185 (84.09)	
Yes	39 (13.59)	35 (15.91)	
RCA, *n* (%)			0.893
No	183 (63.76)	139 (63.51)	
Yes	104 (36.24)	81 (36.82)	
non-culprit CTO, *n* (%)			0.852
No	261 (90.94)	199 (90.45)	
Yes	26 (9.06)	21 (9.55)	
Number of stents, *n* (%)			0.480
1	250 (87.11)	184 (83.64)	
2	35 (12.2)	33 (15)	
3	2 (0.7)	3 (1.36)	
No-reflow, *n* (%)			0.878
No	204 (71.08)	155 (70.45)	
Yes	83 (28.92)	65 (29.55)	
*Intraoperative medication*			
Tirofiban, *n* (%)			0.653
No	224 (78.05)	168 (76.36)	
Yes	63 (21.95)	52 (23.64)	
Atropine, *n* (%)			0.630
No	268 (93.38)	203 (92.27)	
Yes	19 (6.62)	17 (7.73)	
Dopamine, *n* (%)			0.699
No	269 (93.73)	208 (94.55)	
Yes	18 (6.27)	12 (5.45)	
Nitroprusside, *n* (%)			0.446
No	183 (63.76)	133 (60.45)	
Yes	104 (36.24)	87 (39.55)	
*CMR parameters*			
LVEF, %	49.43 ± 0.37	50.16 ± 0.60	0.272
IS, %	12.06 (5.50–19.18)	12.32 (4.45–21.04)	0.828
CO, L/min	5.16 (4.20–6.01)	5.04 (4.20–5.66)	0.270
LVEDV, mL	119.49 (100.67–137.37)	117.29 (99.81–137.33)	0.817
LVESV, mL	41.70 (34.03–60.47)	40.69 (31.65–59.90)	0.744
GLS, %	11.80 (− 15.20 to − 8.5)	− 12.00 (− 15.20 to − 9.20)	0.685
GCS, %	− 13.20 (− 15.80 to − 10.20)	− 13.45 (− 16.00 to − 10.10)	0.2705
GRS, %	20.40 (14.70–25.00)	20.45 (13.85–25.20)	0.87
CMVO, *n* (%)			0.651
No	174 (60.63)	129 (58.64)	
Yes	113 (39.37)	91 (41.36)	
IMH, *n* (%)			0.909
No	193 (67.25)	149 (67.46)	
Yes	94 (32.75)	71 (33.54)	

*AUC*, area under the receiver operating characteristics curve; *BMI*, body mass index; *SBP*, systolic blood pressure; *DBP*, diastolic blood pressure; *HR*, heart rate; *CAD*, coronary artery disease; *WBC*, white blood cell; *NLR*, neutrophil to lymphocyte ratio; *HGB*, hemoglobin; *PLT*, platelets; *hs-CRP*, high-sensitivity C-reactive protein; *cTnI*, cardiac troponin I; *hsTnT*, high-sensitivity troponin T; *CKMB*, creatine kinase isoenzyme-MB; *NT-proBNP*, N-terminal pro-brain natriuretic peptide; *CREA*, serum creatinine; *UA*, serum uric acid; *CHOL*, total cholesterol; *TG*, triglycerides; *HDL-C*, high-density lipoprotein-cholesterol; *LDL-C*, low density lipoprotein-cholesterol, *Lpa*, lipoprotein a; *LDH*, lactate dehydrogenase; *HbA1c*, hemoglobin A1c; *SBT*, symptom onset-to-balloon time; *D2B*, door-to-balloon time; *pre-AP*, pre-infarction angina; *LAD*, left anterior descending; *LCX*, left circumflex branch; *RCA*, right coronary artery; *non-culprit CTO*, non-culprit chronic total occlusion; *LVEF*, left ventricular ejection fraction; *LVEDV*, left ventricular end diastolic volume; *LVESV*, left ventricular end systolic volume; *IS*, infarct size; *CO*, cardiac output; *GLS*, peak global longitudinal strain; *GCS*, peak global circumferential strain; *GRS*, peak global radial strain; *CMVO*, the cardiac microvascular obstruction; *IMH*, Intramyocardial hemorrhage

### Predictors of ALVR after primary PCI

Univariate analysis indicated that males, older age, the increased level of NLR, hsTnT, GLS, IS, the decreased level of LVEF, and the occurrence of Multivascular disease, CMVO and IMH were strongly correlated with the likelihood of ALVR occurring (all *P* < 0.05) (Table 2). After that, the above variables were brought into multivariable logistic backward stepwise regression analysis to explore the independent prognostic risk factors of NSTEMI patients. Multivariate analysis concluded that age (OR: 1.040; 95% confidence interval (CI): 1.009–1.073), the level of neutrophil to lymphocyte ratio (OR: 4.492; 95% CI: 1.906–10.582), the cardiac microvascular obstruction (OR: 3.416; 95% CI: 1.170–9.970), peak global longitudinal strain (OR: 1.131; 95% CI: 1.026–1.246), infarct size (OR: 1.082; 95% CI: 1.042–1.125) and left ventricular ejection fraction (OR: 0.925; 95% CI: 0.872–0.980) were independent risk factors (Table 3).

**Table 2:** Univariable logistic regression analysis for 3–4 months ALVR in the training group

Variables	OR	95%CI	*P* value
Age, years	1.047	(1.020–1.074)	< 0.001
Sex	0.412	(0.176–0.963)	0.041
BMI, kg/m^2^	1.011	(0.941–1.086)	0.775
SBP, mmHg	1.012	(0.997–1.027)	0.116
DBP, mmHg	1.009	(0.989–1.030)	0.386
HR, times/min	1.011	(0.990–1.032)	0.317
Smoker, *n* (%)	1.172	(0.693–1.981)	0.554
Drinker, *n* (%)	1.157	(0.686–1.952)	0.584
Hypertension, *n* (%)	1.153	(0.664–1.999)	0.613
Diabetes, *n* (%)	1.262	(0.707–2.251)	0.431
History of CAD, *n* (%)	1.247	(0.694–2.242)	0.461
WBC, × 109/L	0.950	(0.867–1.041)	0.273
NLR, %			
< 4.03	Ref	Ref	–
4.03–7.68	1.482	(0.753–2.914)	0.254
> 7.68	3.353	(1.725–6.507)	< 0.001
HB, g/L	0.998	(0.986–1.011)	0.798
PLT, × 109/L	0.997	(0.992–1.002)	0.252
HsCRP, mg/dl	1.004	(0.987–1.021)	0.676
cTnI, ng/L	1.003	(0.987–1.019)	0.720
hsTnT, ng/L	1.00009	(1.000003–1.000176)	0.042
CK-MB, ng/L	1.003	(1.0001–1.0054)	0.039
≤ 135	Ref	Ref	–
> 135	1.713	(0.989–2.969)	0.055
NT-proBNP, pg/mL	1.00006	(0.9999–1.0002)	0.449
CREA, mmol/L	1.013	(0.995–1.033)	0.160
UA, umol/L	1.0001	(0.997–1.003)	0.917
CHOL, mmol/L	1.076	(0.849–1.362)	0.545
TG, mmol/L	1.013	(0.832–1.232)	0.901
HDL-C, mmol/L	0.917	(0.343–2.450)	0.862
LDL-C, mmol/L	1.150	(0.862–1.533)	0.341
Lp(a), mg/L	0.999	(0.998–1.001)	0.369
LDH, U/L	1.00008	(1.000–1.001)	0.738
HbA1c, %	1.086	(0.896–1.315)	0.400
SBT, min	1.001	(0.999–1.002)	0.298
D2B, min	1.005	(0.997–1.012)	0.239
Pre-AP, *n* (%)	0.942	(0.520–1.705)	0.843
Killip class, *n* (%)	1.126	(0.624–2.033)	0.693
History of stent implant, *n* (%)	1.650	(0.625–4.358)	0.312
Multivascular disease, *n* (%)	1.761	(1.006–3.081)	0.047
LAD, *n* (%)	1.183	(0.701–1.996)	0.529
LCX, *n* (%)	1.438	(0.697–2.965)	0.326
RCA, *n* (%)	0.933	(0.540–1.610)	0.803
non-culprit CTO, *n* (%)	2.167	(0.948–4.951)	0.067
Number of stents, *n* (%)	1.555	(0.794–3.046)	0.198
No-reflow, *n* (%)	1.368	(0.780–2.400)	0.274
Tirofiban, *n* (%)	1.119	(0.600–2.084)	0.724
Atropine, *n* (%)	1.650	(0.625–4.358)	0.312
Dopamine, *n* (%)	1.052	(0.362–3.056)	0.925
Nitroprusside, *n* (%)	1.173	(0.685–2.010)	0.561
LVEF, %	0.886	(0.842–0.933)	< 0.001
IS, %	1.089	(1.055–1.125)	< 0.001
CO, L/min	0.863	(0.673–1.107)	0.246
LVEDV, mL	1.001	(0.990–1.012)	0.855
LVESV, mL	1.001	(0.988–1.013)	0.913
GLS, %	1.203	(1.109–1.304)	< 0.001
GCS, %	1.005	(0.935–1.080)	0.896
GRS, %	0.989	(0.947–1.032)	0.610
CMVO, *n* (%)	2.725	(1.595–4.654)	< 0.001
IMH, *n* (%)	2.132	(1.243–3.660)	0.006

*CI*, confdence interval; *ALVR*, adverse left ventricular remodeling; *BMI*, body mass index; *SBP*, systolic blood pressure; *DBP*, diastolic blood pressure; *HR*, heart rate; *CAD*, coronary artery disease; *WBC*, white blood cell; *NLR*, neutrophil to lymphocyte ratio; *HGB*, hemoglobin; *PLT*, platelets; *hs-CRP*, high-sensitivity C-reactive protein; *cTnI*, cardiac troponin I; *hsTnT*, high-sensitivity troponin T; *CKMB*, creatine kinase isoenzyme-MB; *NT-proBNP*, N-terminal pro-brain natriuretic peptide; *CREA*, serum creatinine; *UA*, serum uric acid; *CHOL*, total cholesterol; *TG*, triglycerides; *HDL-C*, high-density lipoprotein-cholesterol; *LDL-C*, low density lipoprotein-cholesterol, *Lpa*, lipoprotein a; *LDH*, lactate dehydrogenase; *HbA1c*, hemoglobin A1c; *SBT*, symptom onset-to-balloon time; *D2B*, door-to-balloon time; *pre-AP*, pre-infarction angina; *LAD*, left anterior descending; *LCX*, left circumflex branch; *RCA*, right coronary artery; *non-culprit CTO*, non-culprit chronic total occlusion; *LVEF*, left ventricular ejection fraction; *LVEDV*, left ventricular end diastolic volume; *LVESV*, left ventricular end systolic volume; *IS*, infarct size; *CO*, cardiac output; *GLS*, peak global longitudinal strain; *GCS*, peak global circumferential strain; *GRS*, peak global radial strain; *CMVO*, the cardiac microvascular obstruction; *IMH*, Intramyocardial hemorrhage

**Table 3:** Multivariate logistic regression analysis for 3–4 months ALVR in the training group

Variables	OR	95% CI	*P* value
GLS, %	1.131	(1.026–1.246)	0.013
IS, %	1.083	(1.042–1.125)	< 0.001
Age, years	1.040	(1.009–1.073)	0.012
NLR, %			
< 4.03	Ref	Ref	–
4.03–7.68	1.922	(0.843–4.385)	0.120
> 7.68	4.492	(1.906–10.582)	0.001
CMVO, n (%)	3.416	(1.170–9.970)	0.025
LVEF, %	0.925	(0.872–0.980)	0.009

*ALVR*, adverse left ventricular remodeling; *CMVO*, the cardiac microvascular obstruction; *GLS*, peak global longitudinal strain; *IS*: infarct size; *LVEF*, left ventricular ejection fraction; *NLR*, neutrophil to lymphocyte ratio

### Construction of nomogram

**Fig. 2:**
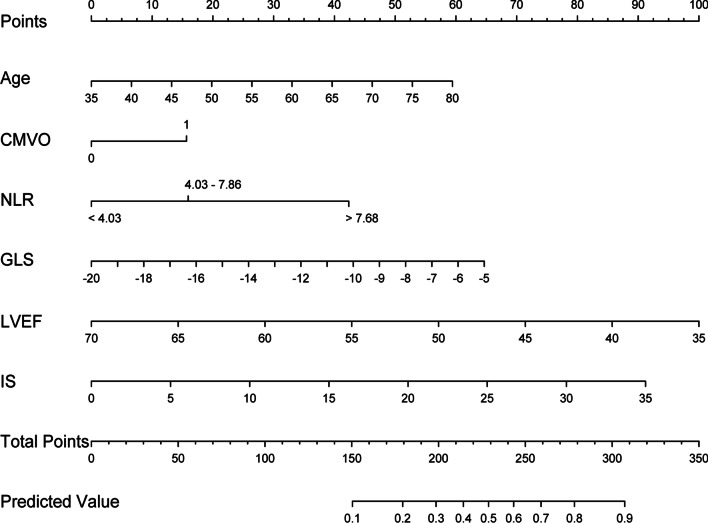
Nomogram for predicting 3–4 months occurrence of ALVR among NSTEMI patients who had PCI. ALVR, adverse left ventricular remodeling; CMVO, the cardiac microvascular obstruction; GLS, peak global longitudinal strain; LVEF, left ventricular ejection fraction; IS, infarct size; NLR, neutrophil to lymphocyte ratio; NSTEMI, non-ST elevation myocardial infarction; PCI, percutaneous coronary intervention

A nomogram model was developed based on six factors (age, NLR, CMVO, GLS, IS, and LVEF) correlated with ALVR, according to the univariate and multiple logistic regression analysis (Fig. 2). Regarding the weight of covariates contained in the model, corresponding points were obtained on the scoring line at the top of nomogram through drawing a vertical line. Finally, the individual probability can be determined on the probability line of ALVR in NSTEMI patients with primary PCI through the total points, which can be calculated by adding the points of six factors together.

### Validation of the nomogram

**Fig. 3:**
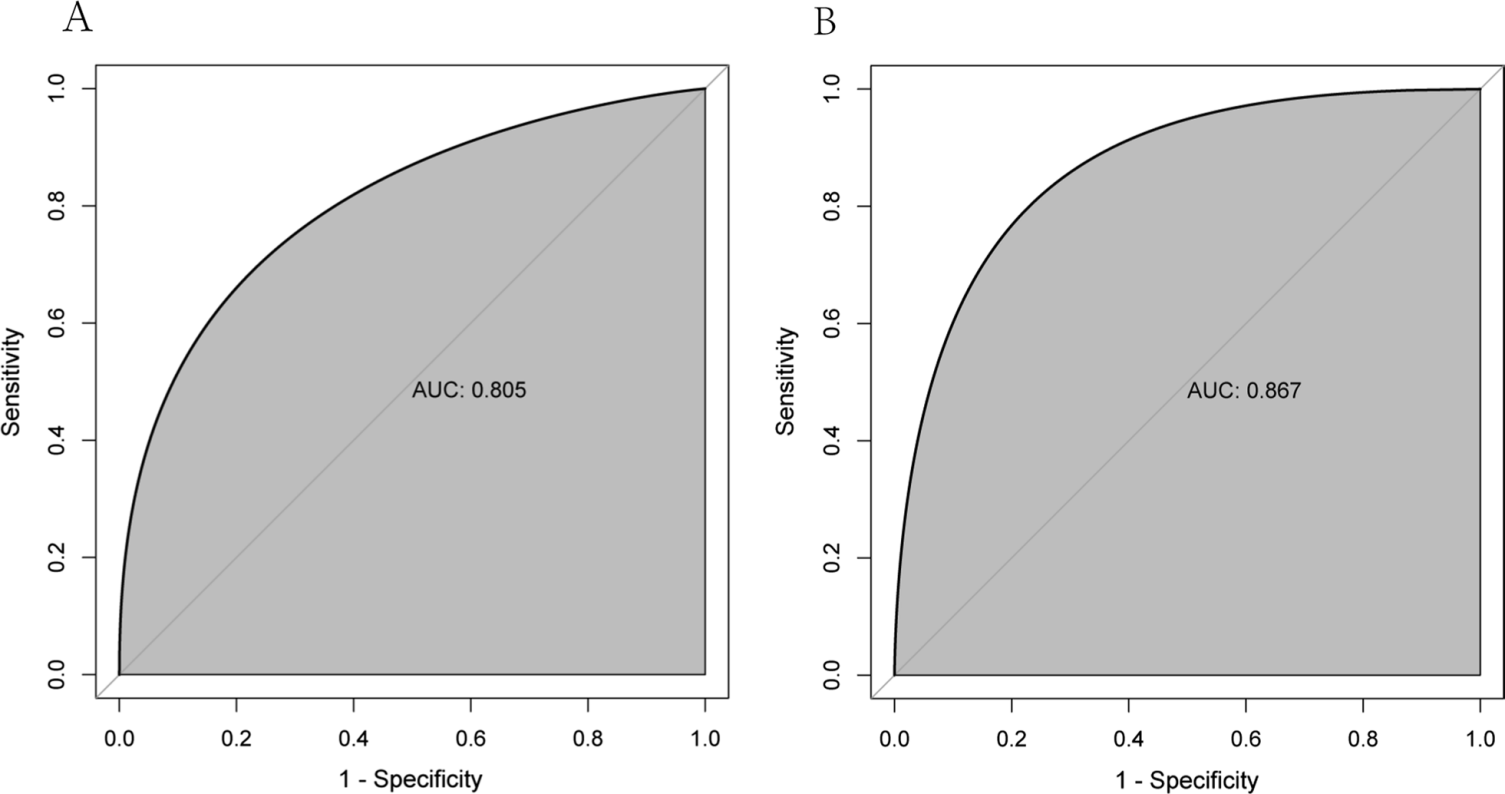
Receiver operating characteristics curve of the nomogram in the training set (**A**) and the external validating set (**B**). AUC, area under the receiver operating characteristics curve

**Fig. 4:**
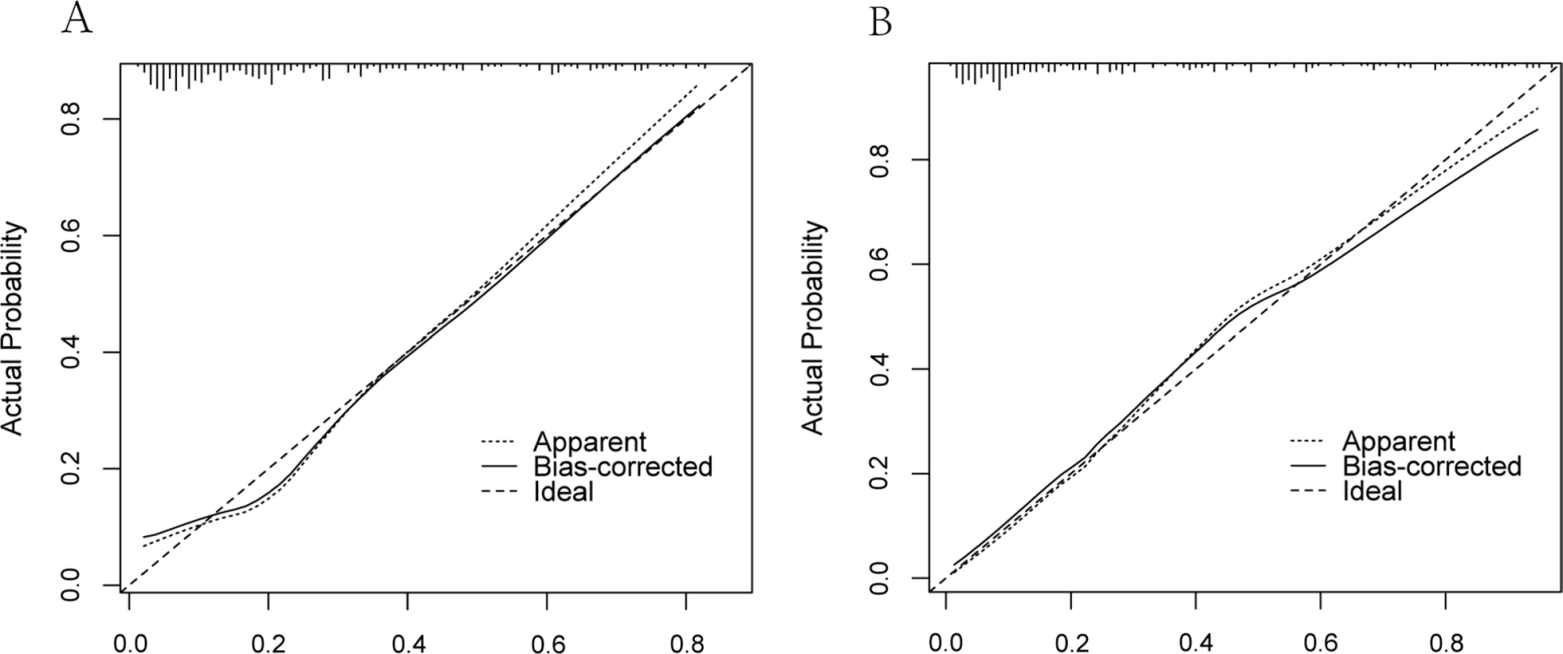
Calibration curve of the nomogram model in the training cohort (**A**) and the external validating cohort (**B**). The x and y axes represent the predicted probability and actual probability, respectively

The area under the curve (AUC) for the nomogram was 0.805 (95% CI: 0.743–0.860) in the development cohort and 0.867 (95% CI: 0.812–0.914) in the test cohort by the receiver operating characteristic (ROC) curve, which demonstrated an outstanding discrimination (Fig. 3A, B). Furthermore, the Hosmer–Lemeshow test proved that the probabilities of ALVR predicted by the nomogram were consistent with the actual occurrence in the training group, the same findings were also obtained from the validation group (all *P* > 0.05). The calibration plots indicated a high consistency between the nomogram (Fig. 4A) and external validation cohort (Fig. 4B).

### Clinical use

**Fig. 5:**
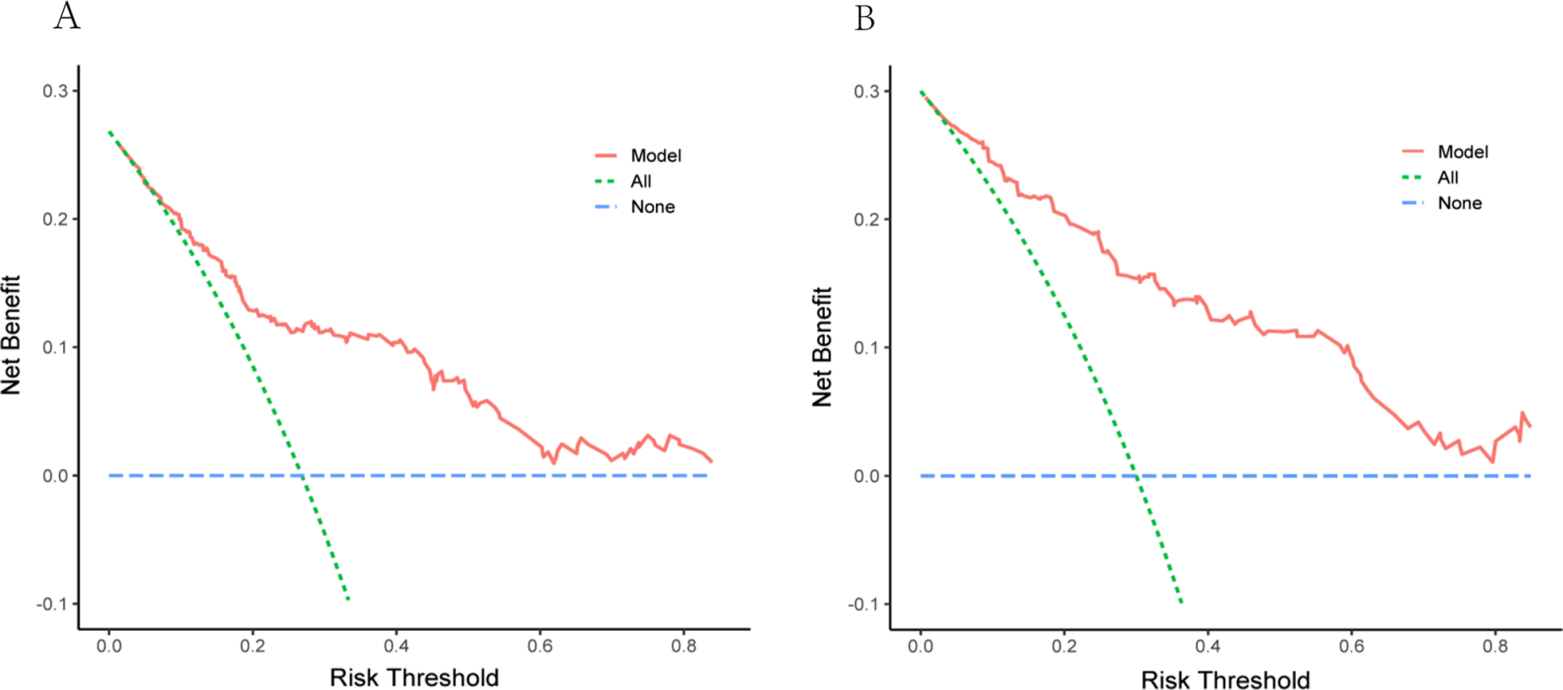
The decision curve analysis for the predicted model in the training group (**A**) and the external validating group (**B**). The y-axis represents the net benefit; the x-axis indicates threshold probability

Decision curve analysis (DCA) was used to appraise the applicability and benefit of the model in two datasets. The nomogram showed superior clinical application value when the threshold probability was between 0.05 and 0.80 in training and external validating cohorts (Fig. 5A, B). During this range, the net benefit of the nomogram model (red line) was higher than that of intervention-all-patients (green line) in predicting the risk of ALVR in NSTEMI patients with primary PCI.

## Discussion

In this study, we aimed to develop and verify a practicable and straightforward tool to predict the probability for the occurrence of ALVR in NSTEMI patients after reperfusion. Based on logistic regression analysis, age, NLR, IS, EF, GLS and CMVO, six factors were ultimately incorporated into the nomogram model, which consisted of cardiovascular risk factor and imaging data. After that, the nomogram showed excellent performance by verification from three aspects: discrimination, calibration, and clinical utility.

Molecular, cellular and interstitial changes during the period from the acute occlusion of coronary artery to ALVR, eventually leading to heart failure [7]. There is a positive association between the severity of ALVR and the risk of death or hospitalization from heart failure [25, 26]. Consequently, early recognition and clinical intervention of ALVR are of great significance for the prevention of structural destruction or functional deterioration. Liu et al. created a risk prediction model for predicting ALVR in patients with acute anterior STEMI, but their study was incomplete, and even some factors that had been shown to be highly associated with ALVR, such as IS and CMVO, were not considered. Besides, the accuracy of related indexes in their study depending on echocardiography was inferior to CMR [15]. In addition, the performance of their model was uncertain due to the lack of not only a validation set, but also tests of calibration and clinical effectiveness.

It is well known that some factors, such as advanced age, IS, and LVEF, have been recognized as risk factors for poor outcomes after AMI [25–27], our results affirmed these conclusions. In epidemiology, the proportion of ALVR after MI in females is higher than in males, and what's more, people with advanced age (female > 65; male > 50) have a high possibility in developing ALVR after MI [26–28]. Wu et al. concluded that the probability of ALVR occurrence could increase considerably if the IS ≥ 18.5%. With advancing age, senescent vascular endothelial cells are capable of weakening vascular function by promoting inflammatory response, oxidative stress and thrombosis [29]. After MI, the abnormal wall movement of ischemic and necrotic segments leads to a decrease in ejection volume. But, remarkably, the distal myocardial segment could generate compensatory motion enhancement; thus LVEF may be still maintained in the normal value to a certain extent and time, resulting in its' poor sensitivity for ALVR prediction. Accordingly, we entered strain-related indicators, a series of parameters that accurately reflect local ventricular myocardial function, into this study.

Extensive studies revealed that strain, correlating with IS and infarct mass, demonstrated independently prognostic values in AMI patients [16, 30–34]. Interestingly, there has been no consensus on which parameter is more valuable in predicting ALVR. A study including 603 MI patients found that both GCS rate and GLS rate measured by echocardiography were the strongly predictive factors of MACE, while only GCS rate could predict ALVR at 20 months (OR: 1.3, 95% CI: 1.1–1.4) [35]. By comparison, another research containing 232 STEMI patients suggested that strain parameters (only GLS) and CMVO determined by CMR were both significantly associated with the ALVR with a follow-up period of 4 months, in agreement with our findings [36]. The inner myocardium is the most sensitive once myocardial ischemia occurs, because the coronary arteries supply blood from the epicardium toward the endocardium. The myocardial fibers beneath the endocardium are mainly arranged longitudinally in the long-axis direction, while GLS mainly reflects the myocardial strain in the long-axis direction. Those mentioned above may explain that when myocardial ischemia occurs, the earliest corresponding change is in the GLS.

ALVR results from the interaction between persistent and dysregulated inflammation and immunoreaction after acute myocardial ischemia. The increase in N count suggests the severity of inflammatory reaction, and the decrease of L count prompts the intensity of stress response [37]. Therefore, the complex factor NLR is capable of reflecting the inflammatory state well and acts ansignificant role in predicting the prognosis in AMI patients. Actually, reperfusion could cause secondary damage to the myocardium, namely ischemia/reperfusion injury, which is in two major forms: CMVO and IMH, and the presence of IMH indicates a more severe degree of microvascular damage [38, 39]. An animal experiment deduced that with the occurrence of CMVO, the myocardial elasticity in the infarcted area decreased, resulting in the increase of local wall stress [40]. Moreover, the CMVO was allied to an increased size of reduced ventricular wall thickness and minor improvement of ventricular wall thickening [41]. The above mechanisms may joint participation in the potential effect of CMVO on ALVR. Our results further corroborated previous reports. It is worth investigating that there is no uniform conclusion on the relationship between IMH and ALVR. Maria et al. [42] concluded that IMH had a positive relationship with the incidence of major adverse cardiac events (MACE), which was detected by T2* mapping. Nevertheless, in Min Jae Cha et al. study, no remarkable discrepancy in IMH (throughT2 sequences) was observed between patients with and without LV remodeling36. In our research, IMH was excluded from the multiple regression analysis, which may be attributed to the fact that only qualitative measurements were performed and the CMR sequences adopted were inconsistent with other studies.

In this study, we speculated that sex, the level of hsTnT, and Multivascular disease had a strong association with ALVR, not the independent risk factors for it. It is not gender but sex hormones that protect the cardiovascular system through neurohumoral regulation. The composition of sex hormones will change along with growing older, and we didn´t carry out an age-adjusted subgroup analysis for gender. As we know, troponin is the biomarker for detecting myocardial injury, and their concentrations change dynamically with with the duration of myocardial ischemia. Partial reports found that peak hsTnT was the marker of the MACE in MI patients undergoing PCI, but the data we collected was at admission, not at peak concentration.

### Limitations

Firstly, the selection bias inherent is inevitable on account of the small sample and retrospective study. Secondly, patients within NSTEMI undergoing primary PCI were not a random sample from the china population. Therefore, it is necessary to perform additional validation of our results with large-scale and multi-center data.

## Conclusions

In this predictive study, the clinical calculating tool provided more customized estimators of the likelihood of ALVR in NSTEMI patients by integrating six independent prognostic factors, including Age, NLR, IS, EF, GLS and CMVO. These estimates contribute to prognostic risk stratification early in clinical management.

## Data Availability

The datasets are available by contacting the corresponding author.

## Abbreviations

ALVR: Adverse left ventricular remodeling
AUC: Area under the receiver operating characteristics curve
BMI: Body mass index
CAD: Coronary artery disease
CHOL: Total cholesterol
CI: Confdence interval
CKMB: Creatine kinase isoenzyme-MB
CMVO: The cardiac microvascular obstruction
CO: Cardiac output
CREA: Serum creatinine
cTnI: Cardiac troponin I
DBP: Diastolic blood pressure
DCA: Decision curve analysis
D2B: Door-to-balloon time
GCS: Peak global circumferential
GLS: Peak global longitudinal strain
GRS: Peak global radial strain
HbA1c: Hemoglobin A1c
HDL-C: High-density lipoprotein-cholesterol
HR: Heart rate
HGB: Hemoglobin
HR: Heart rate
hs-CRP: High-sensitivity C-reactive protein
hsTnT: High-sensitivity troponin T
IMH: Intramyocardial hemorrhage
IS: Infarct size
LAD: Left anterior descending
LCX: Left circumflex branch
LDH: Lactate dehydrogenase
LDL-C: Low density lipoprotein-cholesterol
Lpa: Lipoprotein a
LVEDV: Left ventricular end diastolic volume
LVEF: Left ventricular ejection fraction
LVESV: Left ventricular end systolic volume
NLR: Neutrophil to lymphocyte ratio
non-culprit CTO: Non-culprit chronic total occlusion
NSTEMI: Non-ST elevation myocardial infarction
NT-proBNP: N-terminal pro-brain natriuretic peptide
OR: Odds ratio
PCI: Percutaneous coronary intervention
PLT: Platelets
pre-AP: Pre-infarction angina
RCA: Right coronary artery
SBP: Systolic blood pressure
SBT: Symptom onset-to-balloon time
TG: Triglycerides
UA: Serum uric acid
WBC: White blood cell
